# Editors’ Note

**DOI:** 10.1007/s10202-012-0114-5

**Published:** 2012-11-13

**Authors:** Stephan Lingner, Katharina Mader

**Affiliations:** Europäische Akademie zur Erforschung von Folgen wissenschaftlich-technischer Entwicklungen Bad Neuenahr-Ahrweiler GmbH, Wilhelmstr. 56, 53474 Bad Neuenahr-Ahrweiler, Germany

When Carl Friedrich Gethmann announced *Poiesis & Praxis* in 2001—“A new journal is launched”— this notification came along with high expectations of the addressees with respect to meaning and quality of the content of the newborn journal. The initiative for an “International Journal of Ethics of Science and Technology Assessment” followed the supposed demand for a periodic forum for the rational reflection of the consequences of scientific and technological advance for the individual and social life of the human and its environment—also beyond national perspectives. In the meantime, this broad spectrum was indeed covered with mostly professional articles, which reflected upon the quite different methods of technology assessment and scientific policy advice.

Eleven years, nine volumes, and 36 issues later, the world has changed and with it the journal as well: It came up with new topics and challenges of the scientific-technological civilization but also with new perception modes and reading habits of its addressees. Especially, the rapid development in the field of the neurosciences and hopes for clinical applications were covered by several “Focus” sections accompanied by high public attention. The same held true with respect to the special issues reflecting methodological problems of appropriate science and technology assessment, which had and have to be adjusted to current challenges regarding the relation between technology and society. Finally, during the last few years of *Poiesis & Praxis,* changes of the journal’s publication strategy toward an open access model were successfully acknowledged by both the readers and the authors.

However, the editors are now considering a relaunch of *Poiesis & Praxis* in order to improve its output and impact. By inviting further coeditors to join the journal’s Editorial Board, they want to broaden its perspective—also with respect to its international entitlement. Several renowned representatives of technology assessment in Europe have already expressed their interest. Thus, the issue following the one at hand will present itself with a new appearance. Therefore, the editors of the journal would like to thank the authors and the readers for their interest in *Poiesis & Praxis* so far—by publishing and/or by reading. They also thank the Scientific Advisory Board for its various inputs and critics.

Everyone is welcome to contribute to the journal’s new appearance upcoming in the course of the next year.

Stephan Lingner and Katharina Mader, November 2012

